# Gibberellin-deactivating GA2OX enzymes act as a hub for auxin–gibberellin cross talk in *Arabidopsis thaliana* root growth regulation

**DOI:** 10.1073/pnas.2425574122

**Published:** 2025-07-22

**Authors:** Monika Kubalová, Jayne Griffiths, Karel Müller, Lev Levenets, Edita Tylová, Danuše Tarkowská, Alexander M. Jones, Matyáš Fendrych

**Affiliations:** ^a^Institute of Experimental Botany of the Czech Academy of Sciences, Prague 16502, Czech Republic; ^b^Department of Experimental Plant Biology, Charles University, Prague 12844, Czech Republic; ^c^Sainsbury Laboratory, Cambridge University, Cambridge CB21LR, United Kingdom; ^d^Laboratory of Growth Regulators, Institute of Experimental Botany, Czech Academy of Sciences and Faculty of Science, Palacky University Olomouc, Olomouc CZ-77900, Czech Republic

**Keywords:** gibberellin, auxin, Arabidopsis, root

## Abstract

Plant bodies are built from immobile cells, making the regulation of cell expansion essential for growth, development, and adaptation. In roots, cell elongation executes the movement of the root tips through the soil. This process is tightly controlled by numerous signaling pathways. Among these, gibberellin and auxin signaling stand out for their contrasting effects on root growth, interacting through complex cross talk at multiple regulatory levels. Here, we reveal the molecular basis of the auxin–gibberellin cross talk in the model plant *Arabidopsis thaliana*. We show that the auxin signaling pathway steers the expression of *GIBBERELLIN 2-OXIDASES (GA2OXs)*, key gibberellin-deactivating enzymes in the root elongation zone (EZ). GA2OXs are negative regulators of root cell elongation; *GA2OX8* overexpression decreases gibberellin levels and inhibits root cell elongation; in contrast, the *ga2ox heptuple* mutant roots show elevated gibberellin levels in the EZ and grow longer roots. Intriguingly, shoot-derived auxin can regulate *GA2OX6* and *GA2OX8* expression in roots, linking systemic auxin signaling to local gibberellin level modulation. Together, our findings identify GA2OX6 and GA2OX8 enzymes as key mediators of auxin–gibberellin cross talk, providing insights into their roles in root elongation. These results expand our understanding of how auxin integrates with gibberellin signaling to coordinate root development and growth dynamics.

## Significance

Plant growth and development depend on the precise regulation of cell elongation. The phytohormone gibberellin promotes cell elongation, while elevated auxin levels inhibit root growth. This study reveals that auxin regulates gibberellin levels in *Arabidopsis thaliana* roots by controlling the expression of *GA2OX6* and *GA2OX8* genes, which inactivate gibberellins, thereby limiting cell elongation. *GA2OX* genes act as integrators of gibberellin and auxin signaling. By elucidating this interaction, the study advances our understanding of the mechanisms underlying root growth regulation in plants.

Plants show a remarkable ability to modulate growth in response to developmental and environmental cues. The activity of the root meristem and elongation zone (EZ) is orchestrated by a complex network of phytohormonal signaling pathways to steer the navigation of the root through the soil and to ensure coordinated growth of aerial organs with roots. The phytohormones auxin and gibberellins (GA) play pivotal and sometimes opposing roles in the control of cellular elongation in roots.

The nuclear auxin pathway (NAP) is characterized by the auxin receptor TRANSPORT INHIBITOR RESPONSE1 (TIR1)/AUXIN-SIGNALING F-BOX (AFB), which, in the presence of auxin, associates with coreceptors AUXIN/INDOLE ACETIC ACID (Aux/IAA) proteins, leading to their degradation and the release of AUXIN RESPONSE FACTORS (ARFs) that modulate the transcription of auxin-regulated genes (1). As far back as 1939, it was discovered that auxin inhibits root elongation (2). Surprisingly, a recent study shows that inhibiting NAP initially boosts root cell elongation, and only after several hours, it leads to meristem exhaustion, resulting in inhibition of root growth (3). This highlights the time dependency of auxin responses in the root.

GA are master regulators of cellular elongation (4). GA bind their receptor GIBBERELLIN INSENSITIVE DWARF (GIDs) and DELLA transcriptional repressors (including RGA and GAI), leading to the degradation of DELLA and subsequent activation of GA-regulated gene transcription (5–8). The *Arabidopsis thaliana* root tip exhibits a remarkable gradient of bioactive GA concentration along the primary root axis, rising with distance from the meristem and peaking in the EZ (9–12), which is consistent with GA being positive drivers of cellular elongation. Additionally, GA regulate the cell cycle (13) and control root development (14, 15). The spatial and temporal distribution of GA is determined by a combination of biosynthetic, catabolic, and transport processes (16). To produce bioactive GA, most common precursors GA12 or GA53 undergo a series of reactions mediated by the group of biosynthetic enzymes—GIBBERELLIN OXIDASES—GA20OX and GA3OX (17). Inactivation of bioactive GA is catalyzed by GA2OX enzymes (18). The GA2OX enzyme family in *A. thaliana* consists of nine members: *GA2OX1, GA2OX2, GA2OX3, GA2OX4, GA2OX6, GA2OX7, GA2OX8, GA2OX9,* and *GA2OX10* (18). Overexpression of *GA2OX8* and *GA2OX7* (19, 20) or *GA2OX9* and *GA2OX10* (21) genes leads to reduced levels of bioactive GA and results in dwarfism, delayed flowering, and changed leaf morphology. On the other hand, higher-order loss of function *ga2ox* mutants show elevated levels of active GA compared to the wild type. Consequently, they display phenotypes indicative of GA excess (20–22). Although the importance of GA2OXs in aboveground tissue development is clear, their role in root development is still not understood.

Auxin and GA signaling pathways are interconnected and interact with each other, either positively or negatively during various developmental responses (23). This has been studied at different levels, including their mutual effect on signaling, deactivation, transport, and biosynthesis (24–28).

In Arabidopsis root, GA modulate auxin transport to enhance root responsiveness to auxin (26) or to regulate vascular development (29). Additionally, shoot-derived auxin modulates root growth by regulating the effect of GA response (30). Initial inhibition of NAP increases sensitivity of root to GA (11). Although GA and auxin clearly affect each other during root development, how auxin and GA actions overlap during root cell elongation is not completely understood.

In this work, we focus on two auxin-regulated GA-deactivating enzymes GA2OX6 and GA2OX8. We show that auxin signaling regulates the expression of *GA2OX6* and *GA2OX8* in the root EZ. Consequently, GA2OX6 and GA2OX8 decrease the level of GA, contributing to the regulation of root cell elongation. Our findings reveal the genetic and molecular mechanisms by which the phytohormone auxin regulates the levels of bioactive GA in the root EZ of *A. thaliana*.

## Results

### Auxin Decreases GA Response and GA Levels in Arabidopsis Root Tissue.

**Fig. 1.**
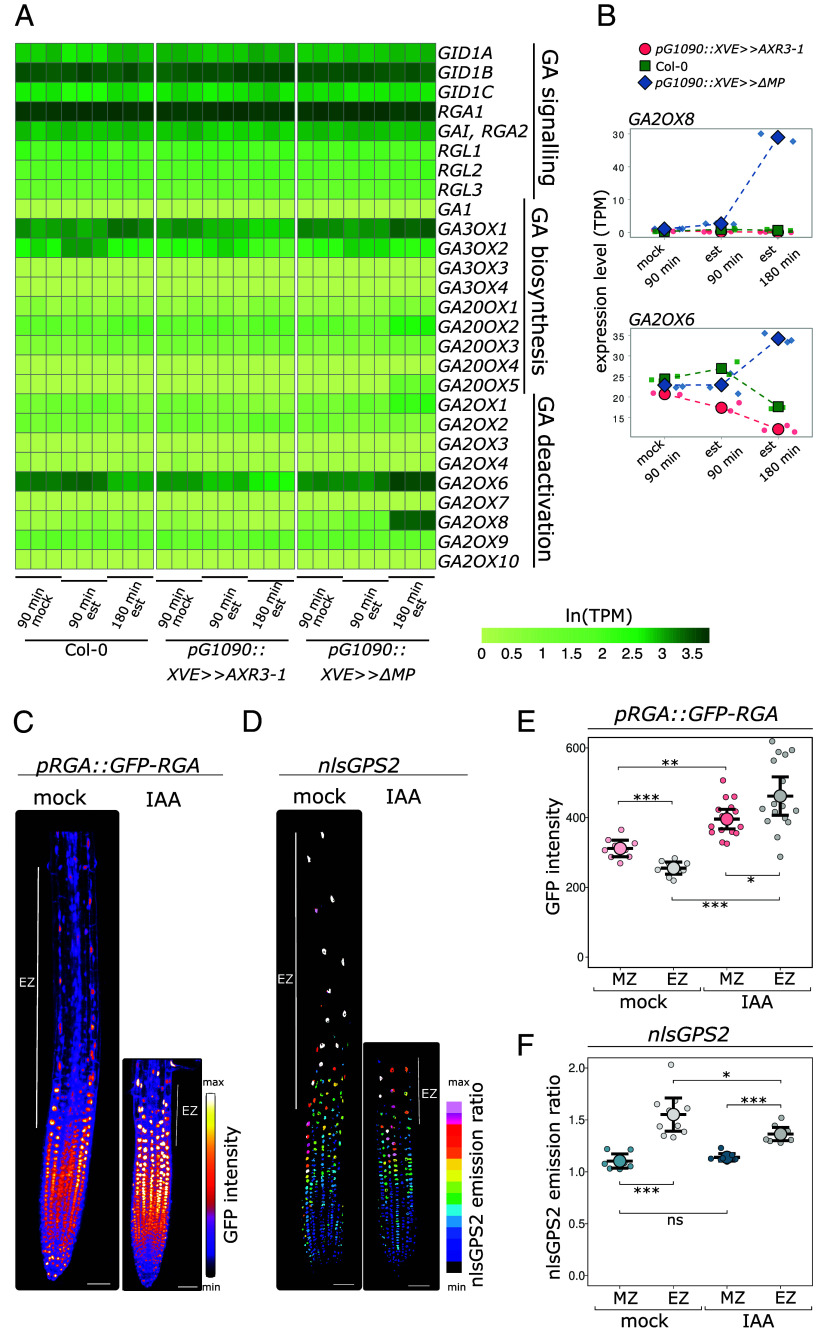
IAA treatment results in a decrease in both the GA response and GA levels in Arabidopsis root tissues. (*A*) Heat map of GA-related genes generated from RNAseq data reflecting ln-transformed TPM (transcripts per million) values in Col-0, *pG1090::XVE ≫ AXR3-1,* and pG1090::XVE ≫ *ΔMP* roots after indicated treatments. (*B*) Relative expression level of *GA2OX6* and *GA2OX8* represented as TPM in *pG1090::XVE ≫ AXR3-1*, *pG1090::XVE ≫ ΔMP* and Col-0 root cells after 90 min of mock or 90 or 180 min of estradiol treatment. (*C*) Root tip of *pRGA::GFP-RGA* treated with 50 nM IAA or mock for 5 h. (*D*) Root tip of *nlsGPS2* treated with 50 nM IAA or mock for 5 h. (*E*) Quantification of GFP-RGA intensity of nuclei in the meristematic zone (MZ) and elongation zone (EZ) of mock- or IAA-treated *pRGA::GFP-RGA* roots. n ≥ 11. (*F*) Quantification of the nlsGPS2 emission ratio of nuclei in the MZ and EZ of mock- or IAA-treated *nlsGPS2* roots. n ≥ 9. The asterisks indicate statistically significant differences based on one-way ANOVA followed by Tukey HSD (*P < 0.05, **P < 0.01, ***P < 0.001). Error bars in boxplots are CI. The EZ is indicated by a white line in *C* and *D*. (Scale bar, 50 μm.)

We have shown previously that by a genetic manipulation of the NAP we can steer the elongation rate of Arabidopsis roots; the inducible overexpression of a dominant inhibitor (dominant *IAA17/AXR3 – pG1090::XVE ≫ AXR3-1*) or activator (dominant *ARF5/MP – pG1090::XVE* ≫ *ΔMP*) of auxin-induced transcription leads to root growth promotion and growth inhibition, respectively (11). We harnessed this system to obtain time-resolved transcriptomic profiles of roots with altered cellular elongation rates (Fig. 1*A*).

Among the differentially expressed genes, we noticed a conspicuous correlation between NAP activity and GA-deactivating genes *GA2OX1, GA2OX6,* and *GA2OX8*. *GA2OX6* and *GA2OX8* showed the strongest auxin signaling-dependent induction among the *GA2OX* family genes. Activation of auxin signaling by the *ΔMP* strongly upregulated both genes while inhibition of the pathway by *AXR3-1* induction led to their downregulation. *GA2OX6* is strongly expressed in the root, and *GA2OX8* shows the strongest upregulation upon activation of NAP (Fig. 1 *A* and *B*). Additionally, the rapid response of *GA2OX6* and *GA2OX8* to auxin treatment in root tips (11) primed them for selection as candidate genes. Two of the GA biosynthetic genes showed a mild upregulation upon activation of auxin signaling but were unchanged in the induced *AXR3-1* roots. The GA signaling genes showed no significant changes (Fig. 1*A* and Dataset S1). This indicates that auxin signaling could lower the levels of active GA in Arabidopsis root tips via activation of GA2OX enzymes that catabolize bioactive GA and its precursors.

Therefore, we analyzed the spatial and temporal dynamics of GA signaling (Fig. 1*C*) and levels of bioactive GA in roots (Fig. 1*D*) upon auxin treatment. We utilized the GA signaling marker line *pRGA::GFP-RGA* (5) in which the GFP signal intensity decreases with activation of GA signaling. To visualize the levels of GA in roots, we used the direct FRET-based nuclear localized *nlsGPS2* (*GIBBERELLIN PERCEPTION SENSOR 2*) biosensor that was recently engineered to be more orthogonal and reversible (10). Both reporter and biosensor lines showed increased GA response (Fig. 1 *C* and *E*) or GA levels (Fig. 1 *D* and *F*) in the root EZ compared to the meristematic zone (MZ), corresponding to the published data (9–12). A 5 h treatment of roots with natural auxin–Indole-3-acetic acid (IAA) led to a significant increase of pRGA::GFP-RGA signal intensity in both MZ and EZ (Fig. 1 *C* and *E*), indicating decreased GA signaling after IAA treatment. The nlsGPS2 biosensor emission ratio decreased after IAA treatment (Fig. 1 *D* and *F*), indicating a decrease in cellular GA levels. Both genetic markers thus show that auxin treatment causes a decrease in GA levels and GA signaling response in the EZ of Arabidopsis roots. The plausible explanation is that this effect is mediated by the activation of *GA2OX6* and *GA2OX8* genes.

### Auxin Signaling Steers the Expression of GA Deactivating Genes *GA2OX6* and *GA2OX8* in the Root EZ.

**Fig. 2.**
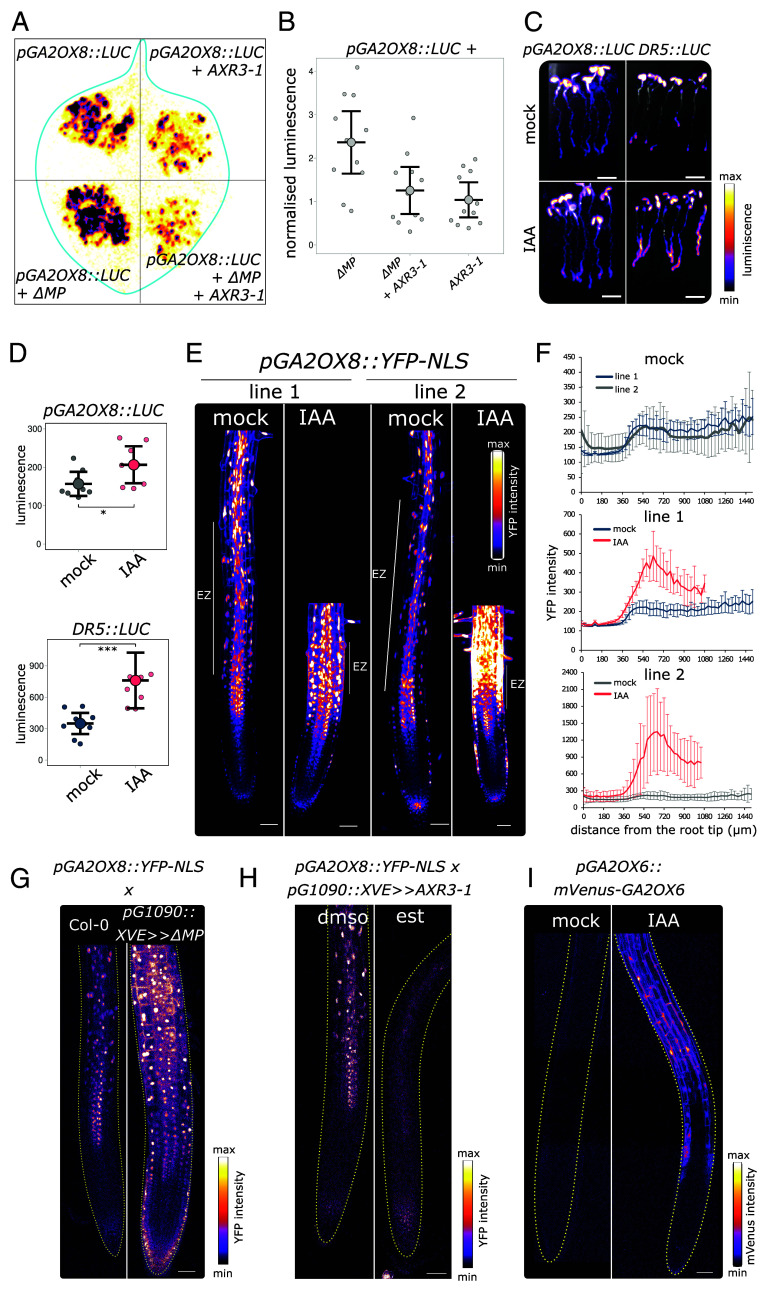
GA-deactivating enzymes GA2OX6 and GA2OX8 are regulated by the auxin–IAA17/AXR3-ARF5/MP pathway. (*A*) Activity *pGA2OX8::LUC* in tobacco leaf cotransformed with or without *p35S::*ΔMP-mScarlet** and *p35S::*AXR3-1-mVenus*.* Representative image shown. (*B*) Quantification of luminescence normalized to *pGA2OX8::LUC*. n ≥ 11 infiltrated leaves. (*C*) Activity of luciferase in *DR5::LUC* and *pGA2OX8::LUC* plants treated with mock or 50 nM IAA for 5 h. (Scale bar, 5 mm.) (*D*) Quantification of luciferase activity in root tips of *DR5::LUC* and *pGA2OX8::LUC* plants treated with mock or 50 nM IAA for 5 h. n ≥ 8. (*E*) Expression of *pGA2OX8::YFP-NLS* of 2 independent homozygous lines in root tips treated with mock or 50 nM IAA for 5 h. The elongation zone (EZ) is indicated by a white line. (Scale bar, 50 μm.) (*F*) Quantification of YFP-NLS intensity along the longitudinal axis of the root in mock- or IAA-treated *pGA2OX8::YFP-NLS* lines. n ≥ 15. (*G*) Expression of YFP-NLS in root tips of *pGA2OX8::YFP-NLS* crossed with *pG1090::XVE ≫ ΔMP* or Col-0, treated for 24 h with estradiol. The outlines of the root are highlighted. (Scale bar, 50 μm.) (*H*) Expression of YFP-NLS in root tips of *pGA2OX8::YFP-NLS* crossed with *pG1090::XVE* ≫ *AXR3-1* treated for 24 h with mock or estradiol. The outlines of the root are highlighted. (Scale bar, 50 μm.) (*I*) Expression of *pGA2OX6::mVenus-GA2OX6* in root tips treated with mock or 10 nM IAA for 90 min. The outlines of the root are highlighted. (Scale bar, 50 μm.) The asterisks indicate statistically significant differences based on Student *t* test (*P < 0.05, **P < 0.01, ***P < 0.001). Error bars in boxplots are CI and in line graphs are SD.

The analysis of *GA2OX6* and *GA2OX8* promoters revealed the presence of AUXIN RESPONSE ELEMENTS (AuxRE) ARF binding sites (*SI Appendix*, Fig. S1*A*). To test whether auxin signaling regulates the activity of the *GA2OX* genes in planta, we first used the transient *Nicotiana benthamiana* expression system to score the effect of *AXR3-1* and *ΔMP* on the activity of the *GA2OX8* promoter. *Luciferase* gene (*LUC*) driven by *GA2OX8* promoter was coexpressed with the dominant inhibitor **AXR3-1-*mVenus* and/or with dominant activator *ΔMP*-*mScarlet* (Fig. 2*A*). The expression of the constructs was verified using confocal microscopy (*SI Appendix*, Fig. S1*B*). *ΔMP* significantly increased the activity of the *GA2OX8* promoter while the coexpression with *AXR3-1* interfered with the promoter activation (Fig. 2 *A* and *B*).

Next, to monitor the activity of the *GA2OX8* promoter on the organ level, we introduced *pGA2OX8::LUC* to Arabidopsis plants, and we observed a strong expression in roots and cotyledons of seedlings. The treatment with IAA led to an increase in LUC luminescence, which was particularly apparent in the younger parts of roots (Fig. 2 *C* and *D*). Similarly, the positive control – *LUC* gene driven by the synthetic *DR5* promoter (31) showed luminescence increase in response to the IAA treatment. Further, we prepared a transcriptional reporter line driving the expression of nuclear-localized YFP (*pGA2OX8::YFP-NLS*). In the root tip, we could detect weak *pGA2OX8::YFP-NLS* signal in the lateral root cap (LRC) cells and the stele tissues of the MZ. A strong expression started in the EZ of the root where the signal was visible both in the stele and the outer tissues epidermis and cortex, and the expression remained high in the root hair zone. The treatment with IAA dramatically increased the *GA2OX8* promoter activity (Fig. 2 *E* and *F*), particularly in the EZ, implying its involvement in the regulation of root cell elongation. Consistently, inhibition of auxin biosynthesis by inhibitor L-kynurenine [L-kyn, (32)] decreased the expression of *GA2OX8* in the EZ (*SI Appendix*, Fig. S1 *C* and *D*).

The expression of *GA2OX8* and *GA2OX6* genes was upregulated by IAA treatment already after 30 min (*SI Appendix*, Fig. S1*E*), hinting at a direct regulation by the NAP. To confirm this, we crossed the *pGA2OX8::YFP-NLS* line with the inducible dominant *pG1090::XVE ≫ AXR3-1* and *pG1090::XVE* ≫ *ΔMP* lines. Induction of irrepressible *ΔMP* led to a significant increase of *GA2OX8* promoter activity in most root tip tissues (Fig. 2*G*). In contrast, the induction of *AXR3-1* almost completely abolished activity of *GA2OX8* promoter in all root tip tissues (Fig. 2*H*).

Finally, we took the advantage of the published *GA2OX6* translational reporter line [*pGA2OX6::mVenus-GA2OX6* (33)]. A weak mVenus-GA2OX6 signal was visible in the epidermis of the EZ and in the vasculature (*SI Appendix*, Fig. S1*F*). Already after 90 min of IAA treatment, the mVenus-GA2OX6 signal increased in the epidermis of the elongation and maturation zone of the root (Fig. 2*I*).

Altogether, these results clearly show that auxin, through NAP´s components IAA17/AXR3 and ARF5/MP, steers the expression levels of *GA2OX8/6* genes in roots and the EZ in particular.

### *GA2OX* Genes Steer Root Cell Elongation.

**Fig. 3.**
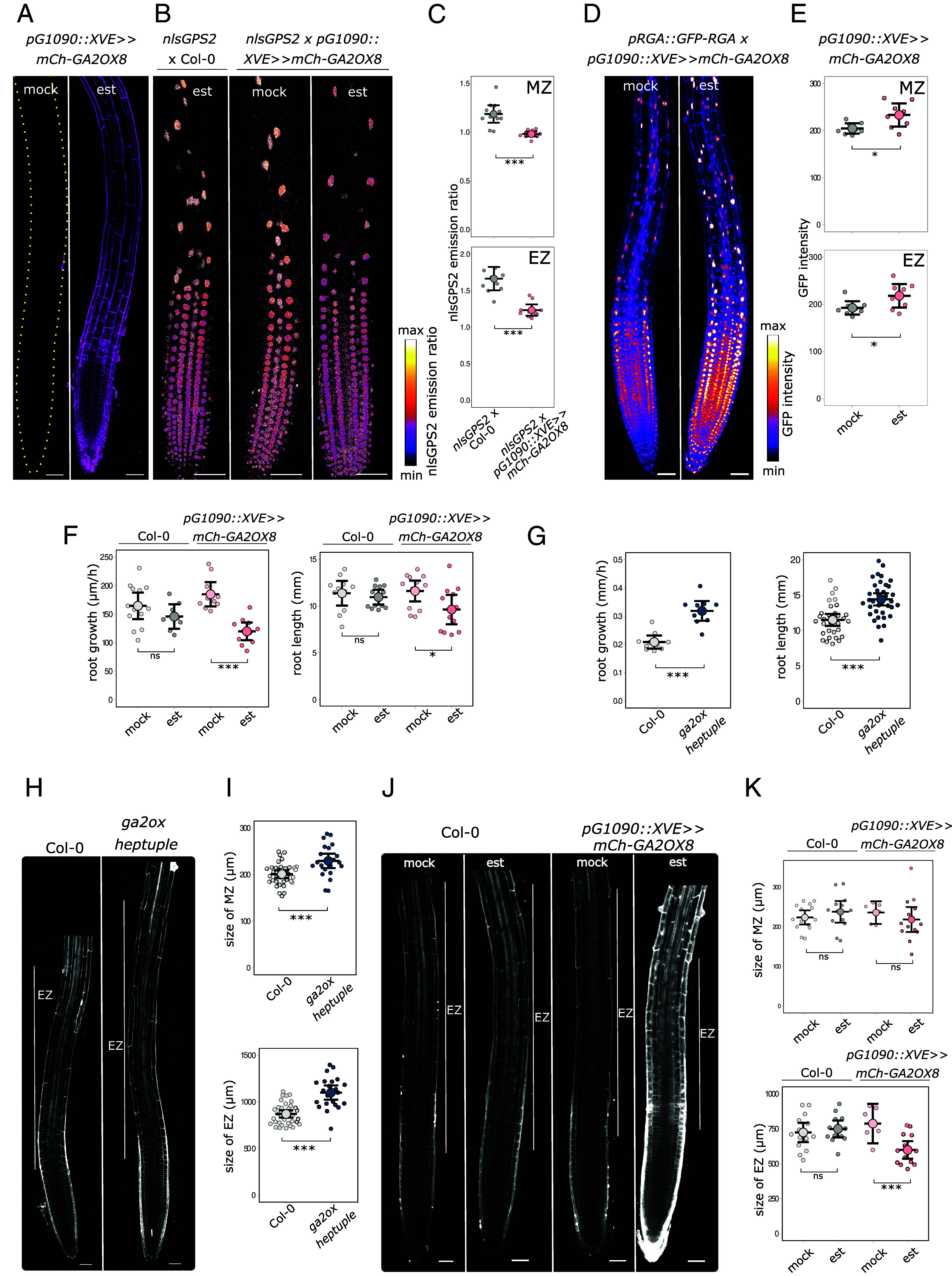
GA2OXs enzymes regulate root cell elongation. (*A*) Root tip of *pG1090::XVE ≫ mCherry-GA2OX8* treated 24 h with estradiol or mock. The outlines of the root are highlighted. (*B*) Root tips of Col-0 x *nlsGPS2* and *pG1090::XVE ≫ mCherry-GA2OX8* x *nlsGPS2* treated for 24 h with estradiol or mock. (*C*) Quantification of the nlsGPS2 emission ratio corresponding to GA levels of nuclei in the meristematic (MZ) and elongation zone (EZ) of *pG1090::XVE ≫ mCherry-GA2OX8* x *nlsGPS2* treated for 24 h with estradiol or mock. n ≥ 10. (*D*) Root tips of *pG1090::XVE ≫ mCherry-GA2OX8* x *pRGA::GFP-RGA* treated for 24 h with estradiol or mock. (*E*) Quantification of the GFP-RGA intensity of nuclei in the MZ and EZ of *pG1090::XVE ≫ mCherry-GA2OX8* x *pRGA::GFP-RGA* treated for 24 h with estradiol or mock. n ≥ 8. (*F*) Quantification of root length of *pG1090::XVE ≫ mCherry-GA2OX8* and Col-0 grown on estradiol and growth rate of *pG1090::XVE ≫ mCherry-GA2OX8* and Col-0 treated 24 h with estradiol. n ≥ 11. (*G*) Quantification of root length and root growth rate of the *ga2ox heptuple* mutant and Col-0. n ≥ 12. (*H*) Root tips of the *ga2ox heptuple* mutant and Col-0 stained with propidium iodide (PI). (*I*) Quantification of the size of the MZ and EZ of the *ga2ox heptuple* mutant and Col-0. n ≥ 22. (*J*) Root tips of *pG1090::XVE ≫ mCherry-GA2OX8* and Col-0 treated for 24 h with estradiol or mock stained with PI. Signal in estradiol-treated *pG1090::XVE ≫ mCherry-GA2OX8* is the combination of PI and mCherry intensity. (*K*) Quantification of the size of the MZ and EZ of *pG1090::XVE ≫ mCherry-GA2OX8* and Col-0 treated for 24 h with estradiol or mock. n ≥ 7. The asterisks indicate statistically significant differences based on one-way ANOVA followed by Tukey HSD and Student *t* test (*P < 0.05, **P < 0.01, ***P < 0.001). Error bars in boxplots are CI. The EZ in *H* and *J* is indicated by a white line. (Scale bar, 50 μm.)

To study the role of GA2OX enzymes in root growth regulation, we analyzed root phenotypes of plants with altered expression of *GA2OX* genes. First, we prepared plants expressing *GA2OX8* from a strong estradiol-inducible system—*pG1090::XVE ≫ mCherry-GA2OX8* (Fig. 3*A*), driving the ubiquitous expression (34). The induction of *GA2OX8* led to decrease of GA levels (Fig. 3 *B* and *C*) and GA response (Fig. 3 *D* and *E*) in both the meristem and the EZ. This indicates that the mCherry-GA2OX8 fusion protein is functional and decreases the level of bioactive GA.

To see the direct effect of *GA2OX8* expression on root growth, we measured root growth rate 24 h after *GA2OX8* induction. Overexpression and accumulation of GA2OX8 led to shorter, slower-growing roots (Fig. 3*F*).

Second, to assess physiological importance of the root-expressed *GA2OX* genes, we analyzed a series of loss-of-function mutants. Root length or root growth rate of single *ga2ox6* or *ga2ox8* T-DNA insertion mutants did not show any significant differences compared to control, the same was true for the *ga2ox6/ga2ox8 double* mutant (*SI Appendix*, Fig. S1*G*). As there are 9 characterized *GA2OX* genes in Arabidopsis, this can be the result of gene redundancy. Therefore, we prepared a mutant in seven *GA2OX1,2,3,4,6,7,8* genes (*ga2ox heptuple* mutant) and analyzed its root phenotype. Mutation in seven *GA2OX* genes led to longer- and faster-growing roots (Fig. 3*G*). Further, we analyzed the longitudinal zonation of *pG1090::XVE ≫ mCherry-GA2OX8* and *ga2ox heptuple* mutant. While mutation of *GA2OX* genes resulted in a longer EZ (Fig. 3 *H* and *I*), overexpression of the *GA2OX8* gene resulted in a shorter EZ (Fig. 3 *J* and *K*). Interestingly, the *ga2ox heptuple* mutant has a slightly longer MZ (Fig. 3*I*). However, overexpression of *GA2OX8* did not induce the opposite alteration (Fig. 3*K*).

To sum up, these results show that GA2OX8 protein decreases the level of bioactive GA in the roots and that this way it regulates root growth predominantly by decreasing the root EZ size.

### Auxin Regulates the Level of GA in the Root EZ Through *GA2OX*s.

**Fig. 4.**
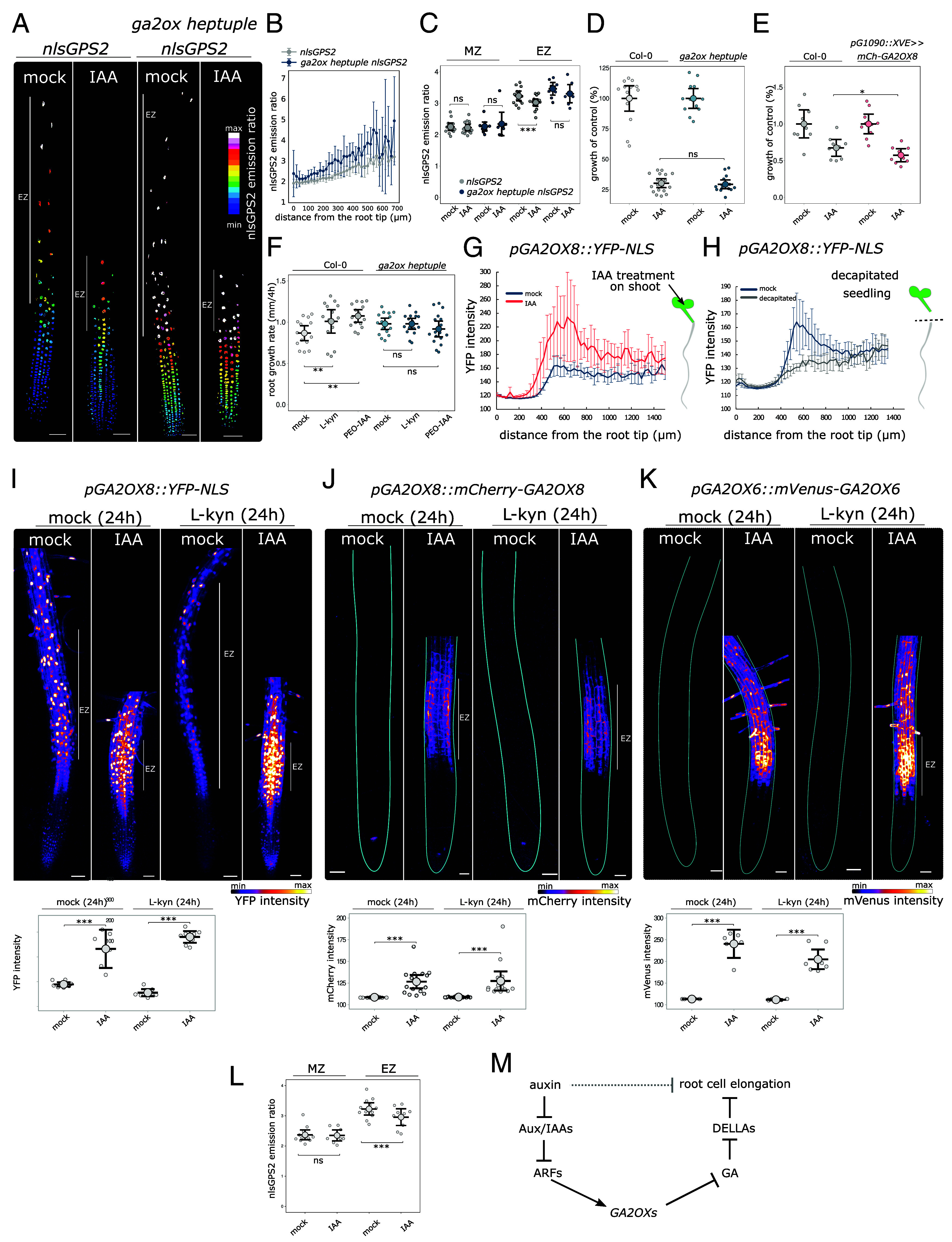
Auxin signaling directly regulates *GA2OX* genes. (*A*) Root tips of *nlsGPS2* and *ga2ox heptuple nlsGPS2* treated for 5 h with 50 nM IAA or mock. (*B*) Quantification of the nlsGPS2 emission ratio corresponding to GA levels along the longitudinal axis of *nlsGPS2*_line1 and *ga2ox heptuple nlsGPS2*_line1 treated for 5 h with 50 nM IAA or mock. n ≥ 10. (*C*) Quantification of nlsGPS2 emission ratio in the meristematic (MZ) or elongation zone (EZ) of *nlsGPS2*_line1 and *ga2ox heptuple nlsGPS2*_line1 treated for 5 h with 50 nM IAA or mock n ≥ 10. (*D*) Growth rate of *ga2ox heptuple* and Col-0 treated with 5 nM IAA for 12 h. Normalized to the mock-treated growth rate. n ≥ 12. (*E*) Growth rate of *pG1090::XVE ≫ mCherry-GA2OX8* and Col-0 treated with 5 nM IAA for 12 h. Normalized to the mock-treated growth rate. All seedlings grown on estradiol. n ≥ 9. (*F*) Growth rate of *ga2ox heptuple* and Col-0 treated with 1.5 μM L-Kyn and 10 μM PEO-IAA for 4 h. n ≥ 14. (*G*) Quantification of YFP-NLS along the longitudinal axis of *pGA2OX8::YFP-NLS* roots in plants treated for 5 h with 100 μM IAA/mock on the shoot. n ≥ 5. (*H*) Quantification of YFP-NLS along the longitudinal axis of *pGA2OX8::YFP-NLS* roots 16h after decapitation. n ≥ 6. (*I*) Root tips of *pGA2OX8::YFP-NLS* pretreated for 24 h with 1.5 μM L-kyn or mock and subsequently treated for 5 h with 100 μM IAA/mock on the shoot. The EZ is indicated by a white line. Quantification of YFP-NLS signal intensity in the EZ. n ≥ 6. (*J*) Root tips of *pGA2OX8::mCherry-GA2OX8* pretreated for 24 h with 1.5 μM L-kyn or mock and subsequently treated for 5 h with 100 μM IAA/mock on the shoot. Quantification of mCherry signal intensity in the EZ. The outlines of the root are highlighted. n ≥ 15. (*K*) Root tips of *pGA2OX6::mVenus-GA2OX6* pretreated for 24 h with 1.5 μM L-kyn or mock and subsequently treated for 5 h with 100 μM IAA/mock on the shoot. Quantification of mVenus signal intensity in the EZ. The outlines of the root are highlighted. n ≥ 5. (*L*) Quantification of nlsGPS2 emission ratio of nuclei in the MZ or EZ of *nlsGPS2* line treated for 24 h with 1.5 μM L-kyn and subsequently treated for 5 h with 100 μM IAA/mock on the shoot. (*M*) A simplified scheme summarizing auxin-modulated regulation of *GA2OX* genes in the root EZ. The asterisks indicate statistically significant differences based on one-way ANOVA followed by Tukey HSD or Kruskal–Wallis test followed by post hoc Dunn’s test (*C*) (*P < 0.05, **P < 0.01, and ***P < 0.001). Error bars in boxplots are CI and in line graphs are SD. The EZ in *A*, *I*, *J*, and *K* is indicated by a white line. (Scale bar, 50 μm.)

The regulation of GA deactivating genes *GA2OX6* and *GA2OX8* by auxin represents a cross talk between these two hormonal pathways. We wanted to address the physiological significance of this cross talk in Arabidopsis roots. First, to test whether auxin directly regulates the GA level through *GA2OX* genes, we analyzed the GA level in the *ga2ox heptuple* mutant after IAA treatment. For this, we transformed the *ga2ox heptuple* mutant with the *nlsGPS2* sensor (Fig. 4*A*). As expected, the *ga2ox heptuple* showed a significantly increased GA level in the EZ (Fig. 4 *A*–*C*), with one of the lines also exhibiting a slightly elevated GA level in the MZ (*SI Appendix*, Fig. S2*C*). Confirming this, liquid chromatography–tandem mass spectrometry analysis of GA metabolites in *ga2ox heptuple* roots revealed elevated levels of the bioactive GA3 and GA19 precursor, while no changes were observed in other GA metabolites (*SI Appendix*, Fig. S2*A*). Further, a qRT-PCR analysis revealed a decrease in expression of *GA20OX2* and *GA3OX1* biosynthesis genes (*SI Appendix*, Fig. S2*B*), again hinting at elevated GA levels in the *ga2ox heptuple* roots that causes a negative feedback on GA biosynthesis (17).

IAA treatment did not cause any changes of GA levels in the MZ, regardless of genotype (Fig. 4 *A*–*C* and *SI Appendix*, Fig. S2*D*). In contrast, in response to IAA, GA levels decreased in the EZ of the control plants, while the *ga2ox heptuple* roots showed a partial resistance to IAA in 2 independent homozygous lines (Fig. 4 *A* and *C* and *SI Appendix*, Fig. S2*D*). In the *ga2ox heptuple* mutants, the nlsGPS2 emission ratio decrease was smaller than in the control (Fig. 4 *A* and *C* and *SI Appendix*, Fig. S2*D*). This suggests that auxin signaling regulates GA levels in the root EZ through *GA2OX* genes.

Further, we tested whether this cross talk can affect the root growth response to auxin. To this end, we treated *ga2ox heptuple* and *pG1090::XVE ≫ mCherry-GA2OX8* line with low concentration of IAA and measured the root growth inhibition rate. Both control and *ga2ox heptuple* roots were inhibited by the same extent (Fig. 4*D*), while the *GA2OX8* overexpression line showed a mild hypersensitivity to auxin (Fig. 4*E*). In Col-0 seedlings, the inhibition of auxin biosynthesis or auxin signaling by PEO-IAA (35) increased root growth rate, while the root growth rate of the *ga2ox heptuple* mutant was unaffected (Fig. 4*F*). In summary, these results show that GA2OXs mediate the cross talk between auxin and gibberellin pathways in the root tips.

### Shoot-Derived Auxin Regulates the Expression of *GA2OXs* in the Root EZ.

Apart from the epidermis, we detected strong expression of *GA2OX6* and *GA2OX8* in the vasculature of the root elongation and maturation zone (*SI Appendix*, Fig. S3*A*). Accordingly, the *ga2ox heptuple* mutant showed increased percentage of xylem vessels compared to control (*SI Appendix*, Fig. S3 *B* and *C*), suggesting that GA2OXs regulate the GA level in the stele, which leads to increased xylem production (36). It was shown that shoot-derived auxin is essential for GA signaling during root growth (25). The vascular expression of *GA2OXs* suggests that their expression might be controlled by the auxin that is transported from the shoot. To test this, we first applied exogenous IAA to cotyledons, which increased the *GA2OX8* promoter activity in the root EZ (Fig. 4*G*). Accordingly, the removal of the shoot as a source of auxin resulted in decreased *GA2OX8* promoter activity (Fig. 4*H*). To rule out the role of local auxin synthesis, we treated the roots with an L-kyn. As expected, auxin biosynthesis inhibition led to a decrease in *GA2OX8* promoter activity; however, auxin application to the shoot led to a strong increase of pGA2OX8::YFP-NLS signal in the EZ (Fig. 4*I*), indicating that the auxin originating from the shoot can regulate the expression of *GA2OX8* in the root. Further, we evaluated the GA2OX6 and GA2OX8 protein levels using translational reporter lines *pGA2OX8::mCherry-GA2OX8* and *pGA2OX6::mVenus-GA2OX6*. Both lines showed weak signals in normal growth conditions. Strikingly, IAA application to the shoot triggered accumulation of both proteins specifically in the EZ, even when endogenous auxin production was inhibited by the L-kyn treatment (Fig. 4 *J* and *K*). Finally, in accordance with the increased *GA2OX6* and *GA2OX8* expression, the levels of active GA visualized by the *nlsGPS2* sensor decreased in the root EZ upon IAA application to shoots (Fig. 4*L*).

In summary, our results demonstrate that auxin signaling can modulate the level of active GA in the root EZ by driving the expression of the *GA2OX* enzymes (Fig. 4*M*). The expression of *GA2OX* genes can be driven also by shoot-derived auxin.

## Discussion

Highly coordinated hormonal regulation is fundamental for root cell elongation (37). This study shows the role of auxin-modulated GA deactivating enzymes GA2OX in root growth and development. An increase in GA levels positively correlates with the root cell elongation rate (9–12). In this work, we show that auxin treatment decreases GA levels and alters the shape of the GA concentration gradient along the longitudinal axis of the root.

Previously, it has been shown that *GA2OX6* and *GA2OX8* genes are specifically regulated by auxin at the whole plant level (24). Interestingly, our results hint at a tight control of GA2OX6 and GA2OX8 protein levels in the roots; while the transcriptomic data and GA2OX8 promoter-reporter line showed a strong expression in roots, the protein levels visualized by fluorescent protein fusions were rather low.

*GA2OX6* and *GA2OX8* overexpression reduced growth of aboveground parts (19, 20, 38); however, their role in roots has not been characterized. Here, we demonstrate that GA2OX6 and GA2OX8 enzymes deactivate GA and are upregulated by auxin treatment in the EZ of the Arabidopsis root. Considering that there is no characterized GA exporter, GA2OX activity can act as main negative regulators of GA levels in the root EZ. Remarkably, the *ga2ox heptuple* mutant showed an increase in GA levels particularly in the root EZ.

What is the biological role of GA2OX enzymes in roots? Both the mutant and the *GA2OX* overexpression data demonstrate that GA2OXs are negative regulators of root cell elongation. The absence of a detectable phenotype in single or double *ga2ox6/ga2ox8* mutants points to gene redundancy, which is remarkable for GA metabolic genes, as overlapping functions have been demonstrated even among genes that show nonoverlapping expression patterns under normal conditions (39). Even the mutation of 5 *GA2OX* genes did not affect GA levels, which corresponds to the unchanged root length and elongation growth of the *ga2ox quintuple* mutant (40). Using the inducible system, we were able to study the direct effect of *GA2OX8* gene induction on root growth while excluding the possible influence of GA level changes on other processes, such as seed germination rate. Additionally, germination is likely regulated by de novo GA biosynthesis, as indicated by the unchanged GA level in *ga2ox quintuple* mutant seeds (22).

The primary driver of GA levels in the root EZ is the activity of biosynthetic enzymes. In addition to differential GA biosynthesis and cellular permeability to GA (40), the deactivation by *GA2OX* genes can jointly regulate GA levels in the EZ. Interestingly, ectopic overexpression of biosynthetic genes and the subsequent increase in GA levels did not lead to enhanced elongation in the early EZ but rather in later tissues (40). This suggests that spatially specific effects of mutating *GA2OX* genes contribute to the regulation of elongation growth.

GA2OX6 and GA2OX8 enzymes were shown to degrade the biologically active GA4 and the GA4 precursor, respectively (20, 41). Our results show that in the *ga2ox heptuple* mutant, bioactive GA3 and the GA19 precursor levels are elevated, while other precursors remain unchanged, indicating a complex metabolic regulation of GA levels.

*GA2OX6* and *GA2OX8* are downstream targets of auxin signaling, representing a cross talk between these phytohormonal pathways. What is the biological significance of this cross talk for root growth regulation? In the *ga2ox heptuple* mutant, the IAA-triggered decrease of GA levels was less significant than in the control. This “molecular phenotype” demonstrates that GA2OX6 and GA2OX8 decrease GA levels in the root EZ in response to increased auxin input. Accordingly, at lowered auxin level or signaling, the *ga2ox heptuple* mutant did not significantly increase root elongation rate. This suggests that auxin–*GA2OXs* cross talk might reinforce the negative auxin effect on cell elongation. In agreement with this hypothesis, the *GA2OX8* overexpressing plants were mildly hypersensitive to external auxin application. However, the control-like sensitivity of *ga2ox heptuple* root to exogenous auxin treatment contradicts this hypothesis. The observed auxin–GA cross talk via *GA2OX6* and *GA2OX8* might be significant only under specific environmental or developmental conditions, which we did not manage to test in our experiments. Our findings suggest that auxin transported from the shoots controls the expression of the *GA2OXs* in the roots. Changes in root elongation growth regulated by shoot-derived auxin may be crucial for adjusting the balance of shoot and root growth under changing environmental conditions. Auxin transported from the aboveground parts, induced by biotic or abiotic stressors (42, 43), could influence root elongation by modulating GA levels.

Additionally, several studies pointed to time-dependent GA response in roots. GA are important in long-term formation of lateral roots (44). In contrast, previously we showed increased sensitivity of roots overexpressing dominant negative *AXR3-1* shortly after altering auxin signaling. Interestingly, these changes are not accompanied by measurable modulations in GA levels (11). This could be explained by increased sensitivity of roots to GA. The positive effect of elevated GA sensitivity on root growth is also demonstrated by the increased root elongation of mutants overexpressing the GA receptor (45).

Remarkably, although the *GFP-RGA* marker line (5) indicated GA signaling decrease in response to auxin both in the meristematic and EZ, the GA level as determined by the direct *nlsGPS2* sensor (10) decreased in the EZ only. This can be explained by higher sensitivity of the *GFP-RGA* signaling reporter, perhaps via the high affinity and root expressed GID1B receptor, compared to the direct *nlsGPS2* sensor that uses GID1C receptor action. Another explanation would be that auxin upregulates expression of *RGA* gene, however in our dataset, *RGA* was not auxin responsive.

In our work, we have focused mostly on the root growth and root EZ. However, the observed strong *GA2OX8* and *GA2OX6* expression in the root vascular system suggests its role during vascular development. Mäkilä et al. showed (36) that GA signaling regulates polar auxin transport to promote xylem production in roots. Our results suggest that *GA2OX* genes are important in vascular organization, as *ga2ox heptuple* mutants favor xylem formation. GA2OX8 or GA2OX6 may catalyze the deactivation of bioactive GA precursors present in vasculature (46) as a part of feedback mechanism to balance vasculature formation.

Our results clearly demonstrate that auxin regulates the GA level in the EZ through upregulation of *GA2OX6* and *GA2OX8* genes and that the GA level modulated by *GA2OX* genes is positively correlated with root cell elongation. However, numerous aspects of auxin–GA interactions in roots continue to be unresolved, with these processes affected by time, specific tissues, hormone concentration, and environmental and developmental factors. Understanding the molecular mechanisms underlying auxin–GA cross talk helps to understand the complexities of root cell elongation.

## Materials and Methods

### Plant Material.

Following lines were used in this article: *pRGA::GFP-RGA* (5), *nlsGPS2* (10), *pG1090::XVE ≫ *A*XR3-1* (47), *pG1090::XVE ≫ ΔMP* (48), *DR5::LUC* (31), *pGA2OX6::mVenus-GA2OX6* (33) and T-DNA insertion mutants *ga2ox6* [SM_3_1859, (22)], *ga2ox8*_line1 (SALKseq_040686.2), *ga2ox8*_line2 [WiscDsLox263B11 (49)].

The following lines were prepared in this study. Above mentioned *ga2ox6* and *ga2ox8* were crossed to create a ga2ox6*/ga2ox8 double* mutant. To generate a *ga2ox heptuple* homozygous mutant containing null alleles of seven *GA2OXs*, the *ga2ox1/2/3/4/6 quintuple* mutant (22) was crossed with the *ga2ox7-2/ga2ox8 double* mutant (SALK_055721/SALKseq_040686). Subsequent generations were then backcrossed with the *ga2ox1/2/3/4/6 quintuple* mutant until the homozygous *ga2ox heptuple* mutant was generated. *pGA2OX8::YFP-NLS* was crossed with *pG1090::XVE ≫ AXR3-1* and pG1090::XVE ≫ *ΔMP*. nlsGPS2 and pRGA::GFP-RGA were crossed with pG1090::XVE ≫ *mCherry-GA2OX8*.

*pGA2OX8::LUC*, *pGA2OX8::YFP-NLS*, *pG1090::XVE ≫ mCherry-GA2OX8, pGA2OX8::mCherry-GA2OX8* were created as described below. *ga2ox heptuple nlsGPS2* was generated by transforming the *ga2ox heptuple* line by the nlsGPS2 plasmid (10) with kanamycin resistance. Primers used for genotyping are in *SI Appendix*, Table S1.

### Growth Conditions.

Chlorine gas was used to surface-sterilize seeds (50), followed by stratification for 2 d at 4 °C. Seedlings were grown vertically on plates containing 1% (w/v) agar (Duchefa) with ½ Murashige and Skoog (MS, Duchefa, 0,5 g/l MES, 1% (w/v) sucrose, pH 5.8 adjusted with 1 M KOH). Plants were grown in a growth chamber with 60% humidity, 22 °C by day (16 h), 18 °C by night (8 h), light intensity of 120 μmol photons m^−2^s^−1^.

### Molecular Cloning and Plant Transformation.

We used GoldenBraid methodology (51) as a cloning strategy. For *GA2OX8* reporter lines, we cloned 1,272 bp upstream of *GA2OX8* (AT4G21200), including 12 bp of its CDS, and fused it with *luciferase* gene (52) (*pGA2OX8::LUC*), YFP fused with NLS (*pGA2OX8::YFP-NLS*) or mCherry with *GA2OX8* CDS (*pGA2OX8::mCherry-GA2OX8*). Next, we cloned CDS of *AXR3-1* (AT1G04250) (88P → L substitution) or 1 to 255 amino acids of *ARF5/MP for ΔMP* (AT1G19850) driven by the *35S* promoter and fused it either with mVenus (3) (*35S::AXR3-1-mVenus*) or mScarlet-I (53) (*35S::ΔMP-mScarlet*) to the C terminus. All these constructs were terminated by *35S* terminator and cloned into the alpha1 vector.

For estradiol-inducible lines, the *G1090* promoter (34), *XVE* (34) and the *Pisum sativum* RuBisCo terminator were cloned into the alpha1-1 vector. The 4xLexA Operon followed by the CaMV *35S* minimal promoter (54), N-terminal *mCherry* (55), *GA2OX8* CDS, and the *35S* terminator, were combined into alpha1-3 vector (56). The alphas were then interspaced with matrix attachment regions (56), combined with a Basta resistance cassette, and cloned into the pDGB3omega1 binary vector (34). All constructs were transformed into the Col-0 ecotype using the floral dip method (57). Primers used for cloning are in *SI Appendix*, Table S2.

### Treatments and Phenotyping.

To treat the plants, 5-d-old plants were transferred to a treatment-containing medium. Estradiol (Sigma, 20 mM stock in DMSO, 2,5 μM working concentration), Indole Acetic Acid (IAA, Sigma, 10 mM stock in 96% ethanol), PEO-IAA (MedChemExpress, 10 mM stock in DMSO), L-kynurenine (L-kyn, Carl Roth, 3 mM stock in DMSO), and propidium iodide (PI, Biotium, 1 M stock in water) were used for treatments. Working concentrations and treatment time for specific experiments are given in the legend of each figure.

Root growth rate was measured as distance between root tip positions in consecutive time frames. The size of the MZ and EZ was measured on roots stained with 2,5 μM PI for 15 min. The end of the EZ was set as the first cell with root hair. The size of the MZ was measured as a distance from the quiescent center to the last isodiametric cortical cell. In auxin-treated roots, it was impossible to determine the meristem size by the cell size rule, as cell elongation is inhibited by auxin. It was shown that the position of the end of the LRC correlates with the end of the transition zone (58). The 5 h IAA treatment did not influence the distance of LRC end from the QC, which was below 300 μm (*SI Appendix*, Fig. S3*D*). Therefore, the boundary of the EZ in Fig. 1 *E* and *F* was defined by the end of the LRC across all lines and treatments, and in Fig. 4*C* and *SI Appendix*, Fig. S2*D*, the EZ boundary was set as the 300 μm distance from the QC across all lines and treatments. To test the role of shoot-derived auxin, shoots were decapitated with a razor. 24 h pretreatment with 1.5 μM L-kyn was used. To add exogenous auxin on shoots, plants were put on agar plates (containing L-kyn or DMSO), and 20 μl of 100 μM IAA treatment was added to cover shoot tissue. To avoid spreading the treatment to roots, 1 to 2 mm gap in the agar was made between the shoot and root.

GA level determination, histological analysis, and qRT-PCR methods are described in detail in the supplemental information.

### Microscopy, Imaging, and Image Analysis.

Vertical stage spinning disk microscope (59) equipped with a Plan-Apo 10x/0.45 M27, Plan-Apo 20x/0.8 M27, and LD LCI Plan-Apo 40x/1.2 Imm objectives were used for most images. The mCherry, mScarlet, and PI-stained samples were excited by 561 nm laser, emission 582 to 636 nm; 515 nm laser for mVenus, Venus, and GFP, emission 520 to 570 nm; and 488 nm laser for YFP samples, emission 500 to 550 nm.

For images 1F, 2G,H, 3B,C, and 4C, Zeiss LSM 880 with LD LCI Plan-Apo 25x/0.8 DIC Corr and C-Apo 40x/1.2Imm Corr DIC objectives was used. In *pGA2OX8::YFP-NLS* crossed with pG1090::XVE ≫ *AXR3-1* or pG1090::XVE ≫ *ΔMP*, GFP and YFP signals were separated using lambda imaging and linear unmixing in the Zen software.

For images 1D, 4A,B, and S2C,D, a Nikon spinning disk microscope (Eclipse Ti-E, inverted) with Yokogawa CSU-W1 SD unit (50 mm) with Plan-Apo L 20x/0.75 objective was used.

Vertically placed flatbed scanner (Perfection V700, Epson) was used for low-resolution imaging. Epson Scanner software v3.9.2.1US was used to acquire images. ImageJ Fiji software (60) was used for all image analysis.

The nlsGPS2 emission ratio was calculated as the ratio of emission intensities of the Aphrodite-FRET acceptor channel (excitation 458, emission 525 to 579 nm) and the Cerulean donor channel (excitation 458, emission 463 to 517 nm). In parallel, the Aphrodite channel was acquired (excitation 514, emission 525 to 579 nm). Intensity of all fluorescent proteins was measured after removing the background intensity only from the nuclei (Figs. 1*F*, 2 *G* and *H*, 3 *B* and *C*, and 4*C*). Ratiometric analysis of nlsGPS2 images (Figs. 1*D* and 4 *A* and *B* and *SI Appendix*, Fig. S2 *C* and *D*) was performed on per-nuclei basis by utilizing the FRETENATOR2 (61) plugin for Fiji. After batch processing of images, each individual label map was manually checked for segmentation quality. Calculation of emission ratios of individual nuclei was performed with local background subtraction to correct for fluorescence of surrounding tissues. To generate the longitudinal profile of nlsGPS2, we used the intensity values of all nuclei from all roots. For the comparison of signal levels between the meristematic and EZ, the mean intensity of all nuclei within each root was calculated and subsequently plotted.

### Transcriptomic Analysis and Gene Expression Analysis.

*pG1090::XVE ≫ AXR3-1*, *pG1090::XVE ≫ ΔMP,* and Col-0 were grown on ½MS plates for 5d and then whole plants were poured with 5 ml of 2,5 μM estradiol-containing ½MS liquid medium. Additionally, *pG1090::XVE ≫ AXR3-1*, *pG1090::XVE ≫ ΔMP* and Col-0 were poured with DMSO-containing ½MS liquid medium. Leftover medium was removed, and plants grown vertically for 1.5 h or 3 h in the cultivation room. After this, roots were harvested. To obtain transcriptomic data, RNA from 40 to 50 root tips (7 to 10 mm) of *pG1090::XVE ≫ AXR3-1*, *pG1090::XVE ≫ ΔMP* and Col-0 was extracted following the protocol (Plant Total RNA Mini Kit, Favorgen). cDNA library was prepared from polyA-enriched total RNA and sequenced by Illumina. The sequencing resulted in at least 20 million 150 bps long read pairs. Rough reads were quality-filtered using Rcorrector and Trim Galore scripts (62). Levels of gene expression (aggregated transcript abundances quantified as transcripts per million—TPM) were determined using Salmon (63) with parameters --posBias, --seqBias, --gcBias, --numBootstraps 30. Reference index was built from *Arabidopsis thaliana*, TAIR10 cds library, version 20101214. Visualization, quality control of data analysis, and determination of differentially expressed genes were selected using sleuth (version 0.29.0) package in R (64). Genes with q-value ≤ 0.05 and log2 fold change ≥ 1 (upregulated) or ≤ −1 (downregulated) were considered to be significantly differentially expressed.

### Luciferase Reporter Assay.

The *pGA2OX8::LUC*, *p35S::*A*XR3-1-mVenus,* and *p35S::ΔMP-mScarlet* constructs were transformed into *Agrobacterium tumefaciens* (GV3101), and used for transient expression in 4-week-old *Nicotiana benthamiana* leaves (65). 1.5d after transformation, leaves were sprayed with 1 mM luciferin, kept for 2 min in darkness, followed by imaging using Azure 600 scanner (6 min exposure; Azure Biosystems). Mean luminescence intensity was determined in each infiltrated region, followed by background subtraction and normalization to the *pGA2OX8::LUC* intensity in the same infiltrated leaf.

### Statistical and Graphical Analysis.

To compare multiple samples with normal distributed data, one-way ANOVA followed by Tukey HSD was used. Kruskal–Wallis test followed by post hoc Dunn’s test was used for data which do not follow normal distribution. Statistical analysis between two normally distributed samples was performed using the unpaired Student's *t* test (*P*-value 0.05 > ns; *P ≤ 0.05; **P ≤ 0.01; ***P ≤ 0.001). All experiments using lines prepared in this study were conducted with at least three independent homozygous insertion lines, with the exception of the *ga2ox heptuple* mutant, which was analyzed using two independent homozygous lines. Each experiment was repeated in at least three biological replicates, and representative results are shown. The statistical test used is indicated in each figure.

Figures were generated using Inkscape. Boxplots were made by SuperPlotsOfData (66). Dots represent individual data points; the largest dot represents mean.

## Supplementary Material

Appendix 01 (PDF)

Dataset S01 (XLSX)

## Data Availability

The original transcriptomic data and the source data for the manuscript have been deposited in Gene Expression Omnibus and Zenodo (GSE282145; https://doi.org/10.5281/zenodo.15689296) (67, 68).
