# Supplementary material for: Gibberellin-deactivating GA2OX enzymes act as a hub for auxin–gibberellin cross talk in Arabidopsis thaliana root growth regulation
Source: Proc Natl Acad Sci U S A. 2025 Jul 22;122(30):e2425574122. doi: 10.1073/pnas.2425574122 (PMC12318176; doi:10.1073/pnas.2425574122)
Supplement: Supplementary file 1 — Appendix 01 (PDF) [file pnas.2425574122.sapp.pdf]

Supporting Information for

"Gibberellin-deactivating GA2OX enzymes act as a hub for auxin-gibberellin crosstalk in *Arabidopsis thaliana* root growth regulation"

Monika Kubalová[1,2*], Jayne Griffiths[3], Karel Müller[1], Lev Levenets[1,2], Edita Tylová[2], Danuše Tarkowska[4], Alexander M. Jones[3], Matyáš Fendrych[1,2,*]

[1]*Institute of Experimental Botany of the Czech Academy of Sciences, 16502 Prague, Czech Republic*

[2]*Department of Experimental Plant Biology, Charles University, 12844 Prague, Czech Republic*

[3]*Sainsbury Laboratory, Cambridge University, CB21LR Cambridge, United Kingdom*

[4]*Institute of Experimental Botany, Czech Academy of Sciences and Faculty of Science, Palacky University Olomouc, Olomouc, CZ-77900, Czech Republic*

*corresponding author names: Matyáš Fendrych, Monika Kubalová

Email: fendrych.m@ueb.cas.cz, kubalovmo@natur.cuni.cz

This PDF file includes:

Figure S1 to S2
Tables S1 to S3
Supplemental methods

Other supporting materials for this manuscript include the following:

Datasets S1

A

Promoter of gene	Position	Strand	Hit Sequence
**GA2OX8** (1272bp upstream)	1238	+	TGTCTc
**GA2OX6** (1750 bp upstream)	164	+	atGTCGGcta
	163	-	aaTGTCGgc
	206	+	TGTCTc
	162	-	taaTGTCGgc
	163	-	aaTGTCGgct

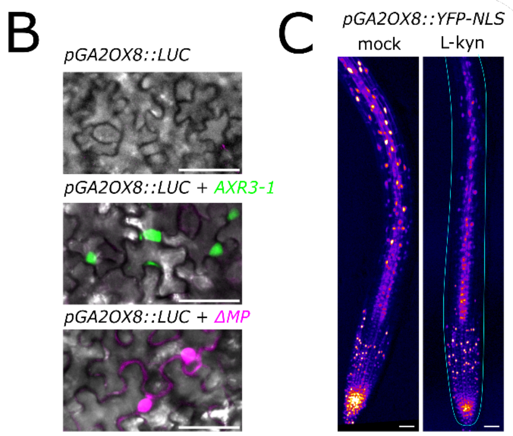

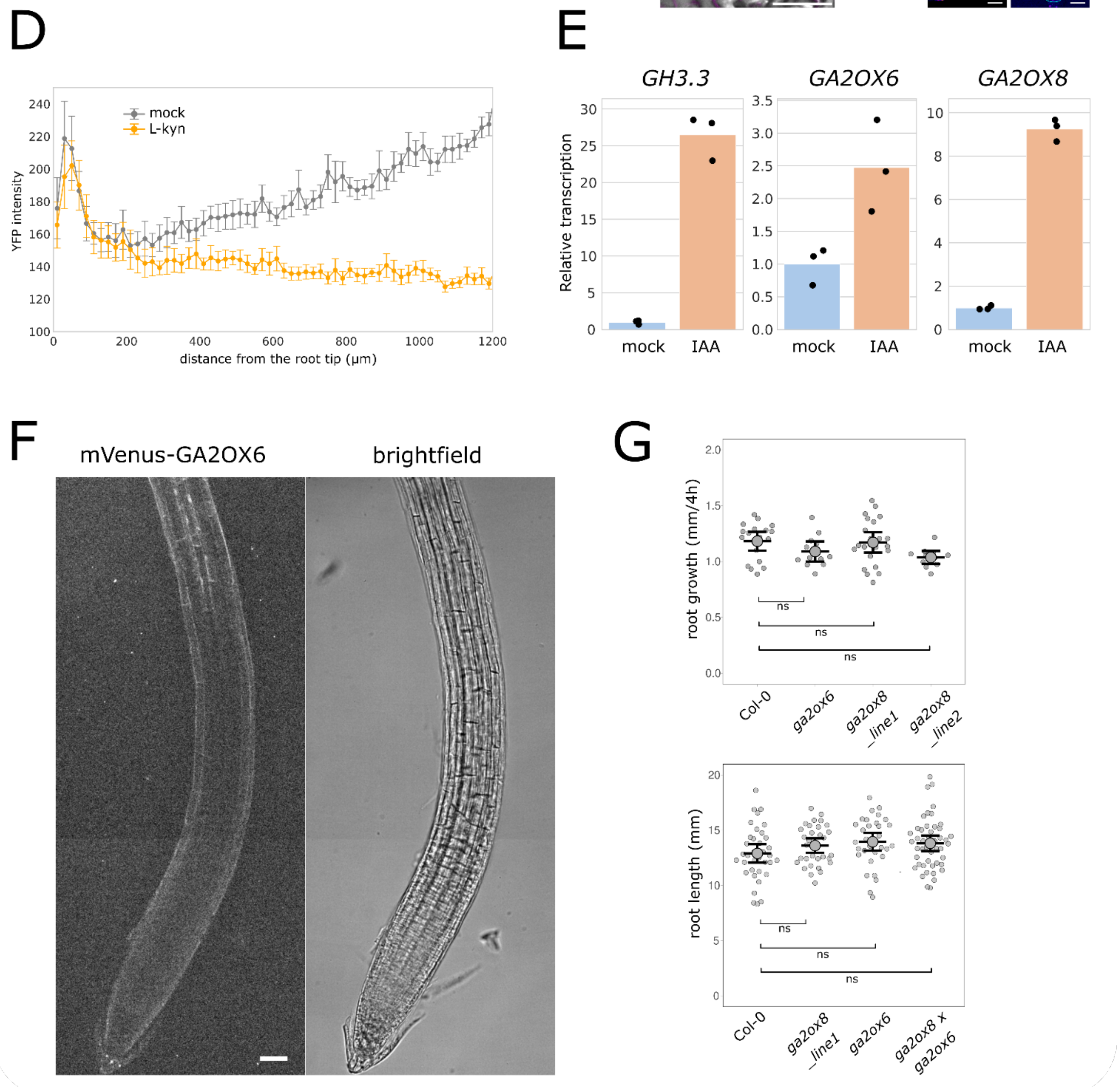

Figure S1

A. AuxRE binding sites in *GA2OX8* and *GA2OX6* promoter (obtained by PlantPAN 4.0).
B. Tobacco leaf cells co-infiltrated with *pGA2OX8::LUC* with/without p35S::*ARF5-mScarlet* and p35S::*AXR3-1-mVenus*. Scale bar = 50µm.
C. Expression of *pGA2OX8::YFP-NLS* in root tip treated with mock or 1.5 µM L-kyn for 24h. Scale bar = 50µm.
D. Quantification of YFP-NLS along the longitudinal axis of the root in mock or L-kyn treated *pGA2OX8::YFP-NLS* line. n≥14.
E. The relative transcription of *GH3.3, GA2OX6* and *GA2OX8* in roots treated with mock or 50nM IAA for 30min.
F. Expression of *pGA2OX6::Venus-GA2OX6* in root tip. Scale bar = 50µm.
G. Root growth and root length of 5d old Col-0, *ga2ox8_line1, ga2ox8_line2, ga2ox6* and *ga2ox8xga2ox6 double* mutant. n≥10.

The asterisks indicate statistically significant differences between groups based on one-way ANOVA followed by Tukey HSD (ns – not significant, *P < 0.05, **P < 0.01, ***P < 0.001). Error bars in boxplots are CI and in line graph are SD.

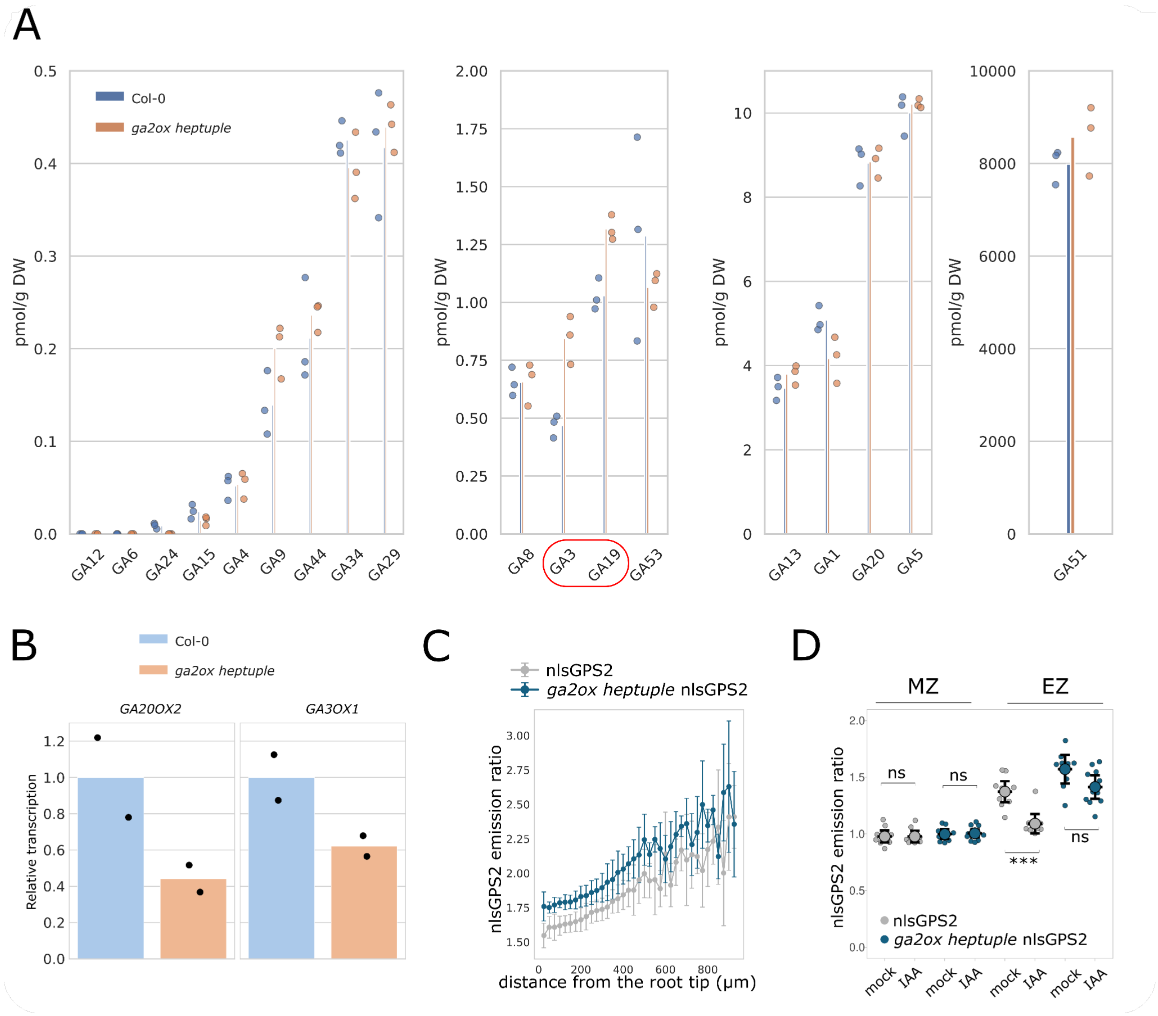

Figure S2

A) Quantification of GA levels in *Arabidopsis thaliana* roots of Col-0 and *ga2ox heptuple* mutant. GA3 and GA19 with detectable differences are highlighted in red.

B) The relative transcription of *GA20OX2* and *GA3OX1* in *ga2ox heptuple* and Col-0 roots.

C) Quantification of nlsGPS2 emission ratio corresponding to GA level of nuclei along the longitudinal axis of nlsGPS2_line2 and *ga2ox heptuple* nlsGPS2_line2 treated for 5h with 50nM IAA or mock. n≥7.

D) Quantification of nlsGPS2 emission ratio corresponding to GA level of nuclei in the meristematic zone (MZ) or elongation zone (EZ) of nlsGPS2_line2 and *ga2ox heptuple* nlsGPS2_line2 treated for 5h with 50nM IAA or mock. n≥8.

The asterisks indicate statistically significant differences between groups based on Kruskal–Wallis test followed by post hoc Dunn's test. (ns – not significant, *P < 0.05, **P < 0.01, ***P < 0.001). Error bars in boxplots are CI and in line graph are SD.

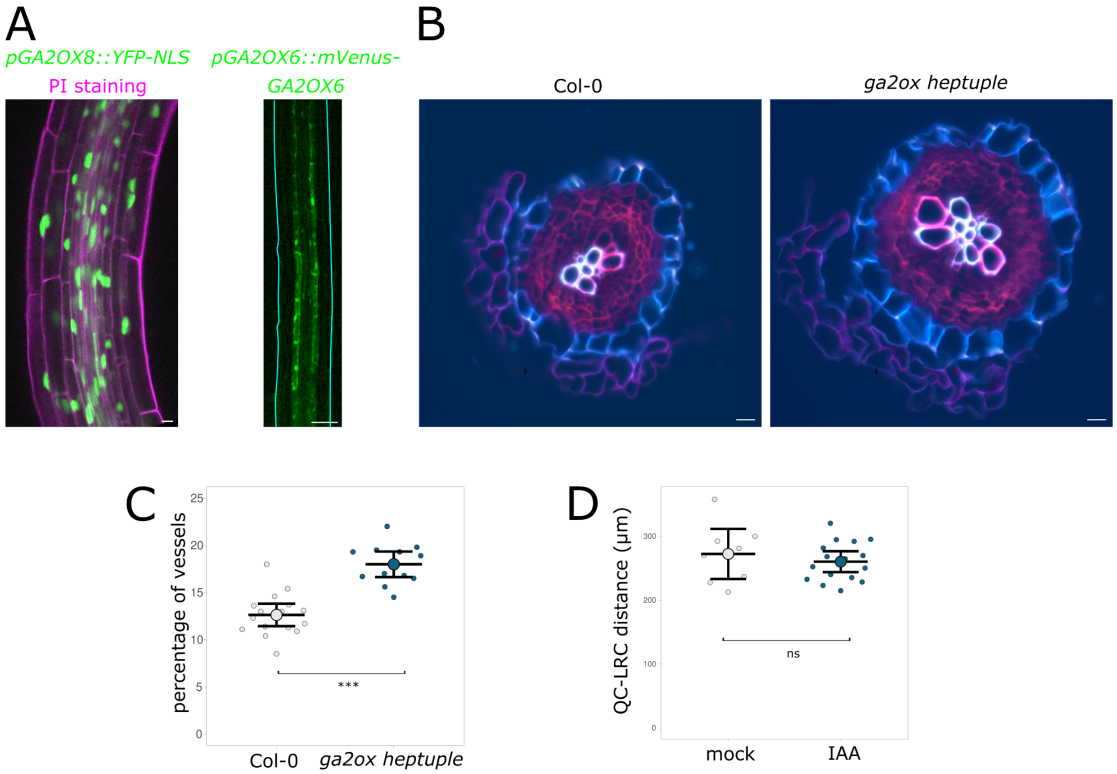

Figure S3

A) Left: Expression of *pGA2OX8::YFP-NLS* (green) in root vascular tissue stained with PI (magenta). Scale bar = 10µm. Right: Expression of *pGA2OX6::mVenus-GA2OX6* (green) in root vascular tissue. The outlines of the root are highlighted with a turquoise line. Scale bar = 50µm.

B) Cross section of *ga2ox heptuple* and Col-0 roots (at the position 0.5 cm from the root base) stained with Safranin O and FastGreen FCF and documented under UV excitation (U-MWU filter block). Scale bar = 10µm.

C) Percentage of differentiated+differentiating vessels in stele of *ga2ox heptuple* and Col-0 roots (percentage of vessels per stele; including vessels of primary and secondary xylem). n≥12.

D) Distance between the quiescent centre (QC) and the end of the lateral root cap (LRC) in Col-0 plants was not affected by the treatment for 5h with etoh (mock) or 50 nM IAA. n≥8.

The asterisks indicate statistically significant differences between groups based on Student t-test (ns – not significant, *P < 0.05, **P < 0.01, ***P < 0.001). Error bars in boxplots are CI.

Supplemental tables

Table S1: List of primers used for genotyping

Primer name	5´-3´ primers	Lines
ga2ox1-1_RP	TCTTCCGGTTCGATATCTCCCA	*ga2ox septuple* mutant
ga2ox1-1_LP	TGACCAAAACACGGACTCGAT	
ga2ox2-1_RP	CTGCGAGGAGTTCGGGTTCTT	
ga2ox2-1_LP	TTTTTGTCGACCCTCCACACC	
ga2ox3-1_RP	TTGAAAATTTGTCCTTTAACCCCA	
ga2ox3-1_LP	TCCGGATGTGAAAACTGAAATCA A	
ga2ox4-1_RP	TGACAGCTCGGCAGTGAATTG	
ga2ox4-1_LP	TGGGGTATCACATTTCACCTCAA	
ga2ox6_2_RP	TTGTCAACCGTATGGAAACCG	
ga2ox6_2_LP	CAACCAAGAACCAACGATTGC	
ga2ox7-2_RP	AGTCACCATGGACTTTCGC	
ga2ox7-2_LP	GAGAAGTGGCGCTAGGGTTT	
ga2ox8_RP	AACGTTCGCAGACTCGTAGT	
ga2ox8_LP	ACCTTTCTTTTGGTTAAGTTACCTT	
ga2ox8_LP	TCGTACCTAGCTTGTTTTTAGAGG	*ga2ox6 x ga2ox8* double mutant
ga2ox8_RP	TGTGGAGAATTATCCCAATAACAA G	
ga2ox6_LP	TTGTCAACCGTATGGAAACCG	
ga2ox6_RP	CAACCAAGAACCAACGATTGC	
SLAT 3' dspm1_LP	CTTATTTCAGTAAGAGTGTGGGGT TTTGG	For ga2ox6
WiscDs-Lox p745_LP	AACGTCCGCAATGTGTTATTAAGT TGTC	For ga2ox1, ga2ox8_line2
SALK LB1.3_LP	ATTTTGCCGATTTCGGAAC	For ga2ox2/3/4/7, ga2ox8_line1
ga2ox8_line1_LP	TCGTACCTAGCTTGTTTTTAGAGG	ga2ox8_line1
ga2ox8_line1_RP	TGTGGAGAATTATCCCAATAACAA G	
ga2ox8_line2_LP	TGAAGTTGACGAAGTTCCTACG	ga2ox8_line2
ga2ox8_line2_RP	GAGCTTCCTGTGATTGACGTC	

Table S2: List of primers used for cloning.

part	5´-3´ primers used for domestication
pGA2OX8	F: GCGCCGTCTCGCTCGGGAGAAGGAACAAAGCAACATTTGGATA<br>R: GCGCCGTCTCGCTCACATTAAAATACGTGTTGTGAGGAGAG
CDS *GA2OX8*	F: GCGCCGTCTCGCTCGTTCGATGGATCCACCATTCAACGAA<br>R: GCGCCGTCTCGCTCAAAGCTTAGTAGACGTGATTAAGGAACC
CDS *AXR3-1*	Part 1<br>F: GCGCCGTCTCGCTCGAATGATGGGCAGTGTCGAGCT<br>R: GCGCCGTCTCGCTGTCTCTGAGAACCCTCTC<br>part 2<br>F: GCGCCGTCTCGACAGTTGATCTGAAGCTAAATCTG<br>R: GCGCCGTCTCGTAAGACGTAAACGCTTGCATG<br>part 3<br>F:<br>GCGCCGTCTCGCTTATGAAAGGATCGGATGCCATTGGTCTTGCTCCGAGG<br>R: GCGCCGTCTCGCTGTCTCTGAGAACCCTCTC
CDS *MP*	Part 1<br>F: GCGCCGTCTCGCTCGAATGATGGCTTCATTGTCTTGTG<br>R: GCGCCGTCTCGGGGACCCGCATATCGCCTTA<br>part 2<br>F: GCGCCGTCTCGTCCCAGCTCTCAGTTGGTAT<br>R: GCGCCGTCTCGTAAGACCGTTCAACTGAGTGT<br>part 3<br>F: GCGCCGTCTCGCTTAAGTTTGACCAGTTCAGTC<br>R: GCGCCGTCTCGCTCACGAACCTGAAACAGAAGTCTTAAGATCGTT<br>for ΔMP<br>F: GCGCCGTCTCGCTCGAATGATGGCTTCATTGTCTTGTG<br>R: GCGCCGTCTCGCTCACGAACCACCGACCATAAGTTGTGACTT

Table S3: List of primers used for qPCR

Gene	AGI number	5´-3´ primers
GA20OX2	AT5G51810	F:AGAAACCTTCCATTGACATTCCA
		R:AGAGATCGATGAACGGGACG
GA3OX1	AT1G15550	F:GATCTCCTCTTCTCCGCTGCT
		R:GAGGGATGTTTTCACCGGTG
EF-1a	AT5G60390	F:TGAGCACGCTCTTCTTGCTTTCA
		R:GGTGGTGGCATCCATCTTGTTACA
GH3.3	AT2G23170	F:TCGGGTTGCGGATAAGTCGA
		R:ACACACCTCGGCACCTTGTA
GA2OX6	AT1G02400	F:GATCCTTTCAAGTTCAGCTCGG
		R:TCTAACCGTGCGTATGTAATCATTC
GA2OX8	AT4G21200	F:CATGGAGCAATGGCATGTACA
		R:GGTTCGTCATCACACGGTGTT

Supplemental methods

Quantitative reverse transcription PCR

Quantitative reverse transcription followed by real-time PCR was performed using DB RT-PCR SYBR Mix (Diana Biotechnologies) at 58°C annealing temperature on a LightCycler480 instrument (Roche). 100-times diluted DNAse-treated RNA was used as a template. Arabidopsis *EF1a* and *ACTIN2* (Arabidopsis gene IDs At5g60390 and AT3G18780, respectively) were used as references. Relative transcript abundancies were then calculated using the equation:

$$rel.transcript\ level = \frac{\sqrt[2]{eff_{ref1}^{CP_{ref1}} . eff_{ref2}^{CP_{ref2}}}}{eff_{target}^{CP_{target}}}$$

Where eff_{ref} and eff_{target} stand for the RT-qPCR efficiencies of reference and target genes, respectively, and CP_{ref} and CP_{target} stand for the respective crossing points of reference and targets genes. Positive transcript levels and the quality of RT-qPCR products were verified by melting curve analysis. Primer sequences are shown in Supplemental Table S3.

Gibberellin level analysis

Gibberellin content was determined in 10 mg of freeze-dried Arabidopsis root samples using three technical replicates per genotype according to the modified method described in Urbanová et al.[1]. Briefly, after adding 1 mL of ice-cold 80% acetonitrile containing 5 % formic acid as extraction solution, the tissue samples were homogenized to a fine consistency using 2.7-mm ceria stabilized zirconium oxide beads (Next Advance Inc., USA) and an MM 400 vibration mill at a frequency of 27 Hz for 3 min (Retsch GmbH & Co. KG, Germany). The crude extracts were then extracted overnight at 4 °C using a benchtop laboratory rotator Stuart SB3 (Bibby Scientific Ltd., UK) after adding internal gibberellins standards ([2H2]GA1, [2H2]GA4, [2H2]GA9, [2H2]GA19, [2H2]GA20, [2H2]GA24, [2H2]GA29, [2H2]GA34 and [2H2]GA44) purchased from OlChemIm, Czech Republic. The homogenates were centrifuged at 36 670 g and 4 °C for 10 min, and corresponding supernatants were further purified using mixed-mode SPE cartridges (Waters, Ireland) and analyzed by ultra-high performance liquid chromatography-tandem mass spectrometry consisted of Acquity UPLC I-Class System (Waters, USA) and triple stage quadrupole mass spectrometer Xevo TQ-XS (Waters, USA) equipped with electrospray interface working in negative mode. GAs were detected using multiple-reaction monitoring mode of the transition of the ion [M–H]- to the appropriate product ion. Masslynx 4.2 software (Waters, USA) was used to analyze the data and the quantitation of GA levels was performed based on the standard isotope dilution method[2].

Microtome sections and histology

Plants were grown for 14d on ½ MS plates. Root anatomy was analysed 0.5 cm from the root base. Root segments were fixed in 4% formaldehyde and embedded into paraffin after dehydration in an ethanol-butanol series[3]. Sections (10µm, Leica 2155 microtome) were collected on alum gelatine-coated slides[3], stained with Safranin O and counterstained with Fast Green FCF[4]. Stained sections were mounted in Pertex and documented under brightfield and UV excitation (U-MWU filter block) on an Olympus BX51 microscope (Olympus Corp., Tokyo, Japan) equipped with a Nikon Digital Sight 10 camera (Nikon Instruments Inc.). Image analysis was performed in NIS Elements 3.22.15 (Laboratory Imaging). Xylem vessels with at least partially lignified cell wall were considered as differentiated, secondary xylem vessels without a thick cell wall, but distinguishable by cell size, were counted as differentiating, and the total area of the stele included the developing phellogen.

References

1. Urbanová, T., Tarkowská, D., Novák, O., Hedden, P. & Strnad, M. Analysis of gibberellins as free acids by ultra performance liquid chromatography-tandem mass spectrometry. *Talanta* **112**, 85–94 (2013).
2. Rittenberg, D. & Foster, G. L. A new procedure for quantitative analysis by isotope dilution, with application to the determination of amino acids and fatty acids. *J. Biol. Chem.* **133**, 737–744 (1940).
3. Soukup, A. & Tylová, E. Essential methods of plant sample preparation for light microscopy. *Methods Mol. Biol.* **1992**, 1–26 (2019).
4. Ohansen, D. A. *Plant Microtechnique*. (McGraw-Hill Book Company, Inc. New York and London, 1940).
